# Multi-Dimensional Modeling of Cerebral Hemodynamics: A Systematic Review

**DOI:** 10.3390/bioengineering11010072

**Published:** 2024-01-11

**Authors:** Jana Korte, Ehlar Sophie Klopp, Philipp Berg

**Affiliations:** 1 Research Campus STIMULATE, University of Magdeburg, 39106 Magdeburg, Germany; 2 Department of Fluid Dynamics and Technical Flows, University of Magdeburg, 39106 Magdeburg, Germany; 3 Department of Medical Engineering, University of Magdeburg, 39106 Magdeburg, Germany

**Keywords:** brain-supplying arteries, circle of Willis, computational fluid dynamics, intracranial aneurysm, medical imaging, multi-scale coupling, 0D modeling, 1D modeling, 3D modeling

## Abstract

The Circle of Willis (CoW) describes the arterial system in the human brain enabling the neurovascular blood supply. Neurovascular diseases like intracranial aneurysms (IAs) can occur within the CoW and carry the risk of rupture, which can lead to subarachnoid hemorrhage. The assessment of hemodynamic information in these pathologies is crucial for their understanding regarding detection, diagnosis and treatment. Multi-dimensional in silico approaches exist to evaluate these hemodynamics based on patient-specific input data. The approaches comprise low-scale (zero-dimensional, one-dimensional) and high-scale (three-dimensional) models as well as multi-scale coupled models. The input data can be derived from medical imaging, numerical models, literature-based assumptions or from measurements within healthy subjects. Thus, the most realistic description of neurovascular hemodynamics is still controversial. Within this systematic review, first, the models of the three scales (0D, 1D, 3D) and second, the multi-scale models, which are coupled versions of the three scales, were discussed. Current best practices in describing neurovascular hemodynamics most realistically and their clinical applicablility were elucidated. The performance of 3D simulation entails high computational expenses, which could be reduced by analyzing solely the region of interest in detail. Medical imaging to establish patient-specific boundary conditions is usually rare, and thus, lower dimensional models provide a realistic mimicking of the surrounding hemodynamics. Multi-scale coupling, however, is computationally expensive as well, especially when taking all dimensions into account. In conclusion, the 0D–1D–3D multi-scale approach provides the most realistic outcome; nevertheless, it is least applicable. A 1D–3D multi-scale model can be considered regarding a beneficial trade-off between realistic results and applicable performance.

## 1. Introduction

The circle of Willis (CoW) is the main neurovascular artery system supplying the brain with oxygen and nutrients. The CoW has three main supplying arteries connecting the anterior and posterior blood circulation: the left and right anterior internal carotid arteries and the posterior basilar artery [1]. These ascend from the aortic arch and combine from the common carotid arteries’ anterior and the left and right posterior vertebral arteries. The anatomy of the aorta and the CoW is subject-specific with differences in bifurcations and artery connections. This should be considered when examining the patient-specific blood flow in the CoW, which is crucial for the study of neurovascular diseases. These diseases comprise, among others, stenoses of the vessel and intracranial aneurysms (IAs). IAs are dilations of the neurovasculature, which carry the risk of rupture. IA rupture can lead to subarachnoid hemorrhage with fatal consequences like stroke or death of the patient [2]. Knowledge about the hemodynamics in IAs can provide information about the pathology’s growth and help understand the rupture risk [3,4]. Understanding the pathology is important for clinical applications like early-stage detection and therapy planning, which in the following can reduce fatal consequences. Therapies nowadays mostly comprise minimally invasive endovascular treatment like stenting or coiling [5]. The important hemodynamic information can be assessed non-invasively through medical imaging like magnetic resonance imaging (MRI) or echocardiography [6]. Though these techniques can give realistic in vivo information about the blood flow, high restriction regarding the spatial and temporal resolution exist and no wall-related parameters can be extracted [7]. The use of non-invasive methods, such as in silico numerical modeling, is required here, as the mentioned disadvantages can be overcome by the numerical evaluation of the realistic flow field on the basis of patient-specific data. Different modeling approaches exist to investigate the blood flow through the CoW and the brain-supplying arteries [8,9]. These have a dimensional (D) range from 0D, solving ordinary differential equations (ODEs), to 1D, solving partial differential equations (PDEs), to 3D numerical simulations, solving PDEs in full volumetric space. The numerical analysis of neurovascular pathologies was studied already in 1988 with 0D computer simulations based on animal trials [10] and in 1991 with 1D computer simulations [11]. In recent studies, these low-scale models are often used to supply boundary conditions for 3D approaches. Lower-dimensional simulations provide hemodynamic information on lower cost and real-time periods and thus can serve as boundary conditions for higher-dimensional simulations [12].

Three-dimensional simulations provide the most highly resolved insight into hemodynamics but nevertheless are expensive and time-consuming [13,14]. Therefore, mostly only a small part of the vessel containing the pathology is analyzed [15]. Moreover, since patient-specific real-time flow data are difficult to capture and are therefore rarely available, the surrounding information, which is used as boundary conditions, is often based on assumptions or taken from the literature [16]. In addition to receiving the boundary conditions from low-scale models, coupled multi-scale approaches exist, directly exchanging flow and pressure information [17,18]. Multi-scale coupling carries the advantage of incorporating and exchanging information over the dimensions and models, leading to a more realistic simulation of the patient-specific flow [19]. Challenges regarding the multi-scale analysis of hemodynamics arise considering the individual structure of the CoW anatomy, the complex morphology of the pathologies and the patient-specific as well as posture- and motion-specific pulsatile flow. Moreover, modeling challenges regarding the interaction between different dimensions are a crucial factor in the development of the most realistic blood flow reproduction of neurovascular pathologies. Subsequently, regardless of the wide-ranging development, validation and application of numerical low-, high- and multi-scale models within the past years, up to now no decision on the most realistic and applicable model could be derived. Clinical applicability relies on this decision, which needs further investigation to provide significant results. This paper aims to review the existing approaches and numerical models, focusing on multi-scale modeling of the CoW and the supplying arteries, namely the inflow into the brain circulation. Recent research will be discussed and described accordingly. First, the existing models, comprising 0D, 1D and 3D models, will be discussed. Second, the review of the multi-scale coupling of these models, looking at the 0D–1D, 0D–3D, 1D–3D and 0D–1D–3D coupling, will be performed. The most important findings according to a realistic reproduction of the blood flow and the applicability of the respective approaches will be presented. In the following, the crucial weaknesses of the models regarding these factors will be discussed and the required future steps will be extracted.

## 2. Methods

The literature search was carried out using the keywords “circle of willis hemodynamic blood flow simulation” in the databases PubMed and ScienceDirect. Exclusion of articles was structured based on Atkinson et al. and Moher et al. [20,21].

**Figure 1:**
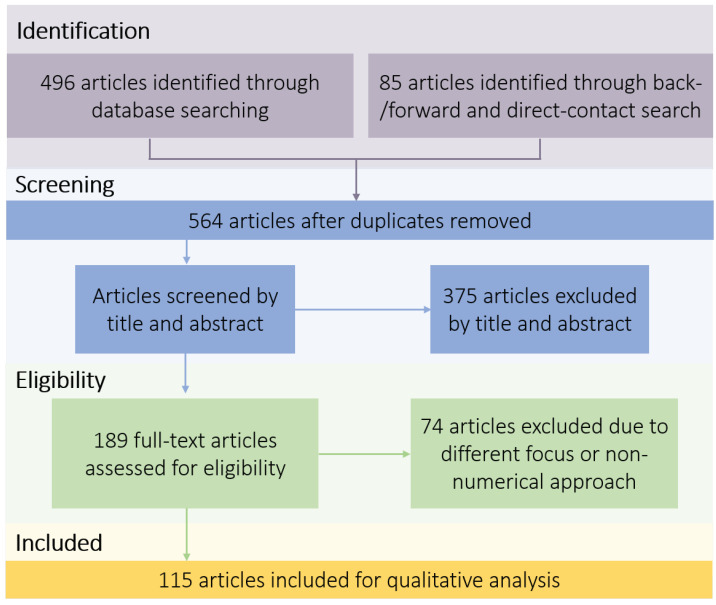
Flowchart describing the underlying study selection process for the performed review analysis based on [20,21].

As described in Figure 1, articles were taken into account within the database search and further extended by a backward (screening of reference list) and forward search (screening of citations) as well as a direct contact search (communication to individual researchers) [21]. After duplicates were removed, this search returned 564 results in total for a first screening. This included a filtering based on title and abstract to determine whether articles could contribute to this work. As such, articles in which the neurovascular system was not mentioned or solely an experimental approach was performed were excluded. In the next step, the full texts of the remaining articles were read. If it became clear while reading that the articles did not contribute to the method development of low-, high- or multi-scale neurovascular hemodynamic analysis, these were also excluded. In total, 115 articles were analyzed in this review, comprising different model dimensions and different multi-scale models. The studies were firstly categorized by the developed methods based on their model dimensions in three categories: 0D, 1D and 3D models. Secondly, the multi-scale models were categorized in four categories: 0D–1D, 0D–3D, 1D–3D and 0D–1D–3D models. Within the former, the models and their applications are presented and discussed. Concerning the latter, a review was conducted on the methods upon which the coupling was based. The review finishes with an evaluation of the models’ benefits and their relations and an outlook on important future work in this field. A summary of chosen articles analyzed within this study is presented in Table 1 to give an overview of representative studies for each of the dimensions (0D, 1D, 3D, multi-scale).

## 3. Mathematical Models

Different spatial scales exist to describe the hemodynamics in the complex cerebrovascular structure numerically. These numerical models entail specific advantages and disadvantages regarding computing hours, provided information and proximity to reality depending on the scale. In the following section, the numerical models based on three spatial scales (0D, 1D, 3D) are introduced and the application for the neurovasculature is reviewed.

### 3.1. Zero-Dimensional Models

**Figure 2:**
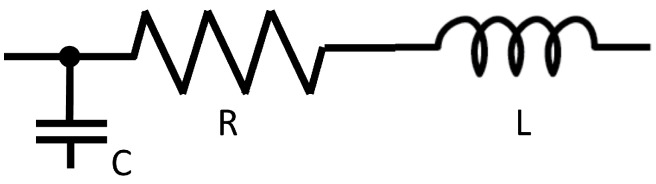
Sample arterial compartment comprising a 3-element Windkessel model with compliance (C), resistance (R) and inductance (L) [136].

Zero-dimensional models are based on the similarity of the uniform distribution of pressure, volume, and flow in the human arteries compared to electrical current in circuits. Here, the vascular system is simplified into compartments describing the numerical connection using Windkessel (WK) equations [1,136]. This makes them valuable tools for studying pressure and flow distribution under specific physiological conditions, providing insights into the overall cardiovascular dynamics. The models follow Kirchhoff’s laws, yielding differential–algebraic equations and allowing efficient simulation of the circulatory system, accounting for the heart, venous system and regulatory dynamics with minimal computational resources [129,136]. Blood flow is described through principles like mass conservation and Poiseuille’s law for steady flow. These principles find an analog in electrical circuits with elements like resistance (R), inductance (L), capacitance (C), voltage (U) and current (I) [136], as presented in Figure 2.

The equations used within the electrical circuit are set as follows:

(1) $${\mathrm{Ohm}}^{\prime }\mathrm{slaw}:I=\frac{\Delta U}{R}\Rightarrow Poiseuille^{’}s\;law:Q=\frac{\Delta P}{R}$$

(2) $$CdP_{in}=\left(Q_{in}-Q_{out}\right)dt$$

(3) $$LdP_{out}=\left(R\cdot Q_{out}\right)dt$$

Values of *R*, *L*, and *C* are determined based on vessel geometry and mechanics. The underlying formulas are presented in the following:

(4) $$R=\frac{8\pi \mu l^{3}}{V^{2}}$$

(5) $$L=\frac{9\rho l}{4\pi r^{2}}$$

(6) $$C=\frac{3\pi lr^{3}}{2Eh}$$

with ρ = fluid density, *l* = vessel length, *r* = vessel radius, *E* = elasticity module, *h* = vessel thickness [33,50,137]. WK models were developed to represent these principles effectively. Initially, the two-element WK model emerged, offering a reasonable portrayal of afterload on the heart and aortic pressure during diastole, although with less accuracy during systole. To address this, a three-element WK model was introduced, and further enhancements led to the four-element WK model [46]. For a more comprehensive understanding of microcirculation dynamics, researchers have explored five-element and six-element WK models. These models describe individual compartments and can be combined to create multi-compartment models, offering flexibility to tailor the models for specific research purposes [136].

**Validation**

One of the first WK computer simulations was implemented by Burkhoff et al. with the model parameters based on an in vivo animal study in 1988 [10]. Ten years later, Burattini et al. used an in vivo study as well to estimate a viscoelastic WK model. Using viscoelasticity optimizes the WK model compared to an elastic one [26]. First validation studies in regard to change in position (sitting to standing) were performed in 2004 by Olufsen et al. [38]. The study showed an increase in hemodynamic pressure and velocity when a position change is performed. This investigation is crucial for finding the true model parameters, since body movement and position change are neglected in conversational studies. Regarding the true WK model parameters, Pope et al. carried out a sensitivity analysis [39]. Here, five parameters were found to be crucial for reliability. Furthermore, the impact of aging was estimated and an increase in cerebral systemic resistance as well as peak systolic ventricular pressure was found. A reduction with aging was found only for arterial compliance. One of the first model reductions was performed by Vande Vosse et al., showing the advantage of lower computing costs but surely showing the disadvantage of accuracy [138].

**Application**

Zero-dimensional models in the field of cerebrovascular research were variously applied to either further develop the models or analyze different pathologies [33]. Abdi et al. investigated the impact of a stenotic vessel in the WK model [22]. A RCL network was used starting from the carotic communicating artery and results were validated with the literature. Zhang et al. focused on the variations in the anatomy of the circle of Willis based on different positions of the subject (supination to standing) [50]. Within this model, they further investigated the impact of a stenosis and validated their results with Doppler ultrasound measurements. Sedraie et al. utilized the model to analyze a stenosis and validation in this case was performed with the literature as well [42]. In contrast, Sethaput et al. investigated the impact of different arterial formations of the CoW on the blood flow with regard to occurring brain injuries [139]. Later on, McConnell et al. created a steady-state model of the neurovascular network and carried out simulations investigating different variations in occlusion and stenosis of the CoW [34]. They found that autoregulation is a crucial factor in collateral flow through the CoW. To optimize the model parameters, Lal et al. captured in vivo data and performed further development of the models [32]. Furthermore, Chnafa et al. validated the model with three-dimensional computational fluid dynamics data and experimental in vitro data [27]. The limitation in this study was the assumption of no energy loss over the bifurcations, which is then named as the crucial discrepancy to reality.

### 3.2. One-Dimensional Models

Using the cylindrical shape of blood vessels, 1D models are created to represent hemodynamic changes along arteries [1,129,140]. These models assume laminar blood flow with an impermeable, sliding wall. Some consider blood seepage into the wall [141,142], but radial and tangential velocities are usually neglected [143,144]. The 1D equations for mass and momentum conservation can be derived from control volumes or by integrating the incompressible Navier–Stokes equations across a cross-section [143]. The equations are presented in the following, with *A*: cross-sectional area, *Q*: flow, *P*: pressure, *t*: time, *z*: vessel length, ρ: density, $K_{R}$: blood resistance [1,114].

(7) $$\frac{\partial A}{\partial t}+\frac{\partial Q}{\partial z}=0$$

(8) $$\frac{\partial Q}{\partial t}+\frac{\partial }{\partial z}\left(\frac{Q^{2}}{A}\right)+\frac{A}{\rho }\frac{\partial P}{\partial z}+K_{R}\frac{Q}{A}=0$$

To solve the system, an equation describing the material properties of the wall (parameters *A* and *P*) is required [1,114,132], with *h*: artery wall thickness, index 0: reference, *E*: elasticity module, σ: Poisson number.

(9) $$P-P_{0}=\frac{Eh_{0}}{r_{0}\left(1-\sigma^{2}\right)}\left(\sqrt{\frac{A}{A_{0}}}-1\right)$$

Regarding the impact of the geometry, namely bifurcations within the vessels, flow conditions at these locations are described with the mass conservation and the overall pressure continuity. The equations are shown in the following, with indices 1 to 3 representing the proximal (1) and distal (2,3) parent arteries and U representing the mean axial velocity [23,85,145,146]:

(10) $$A_{1}U_{1}=A_{2}U_{2}+A_{3}U_{3}$$

(11) $$P_{1}+\frac{1}{2}\rho U_{1}^{2}=P_{2}+\frac{1}{2}\rho U_{2}^{2}+P_{3}+\frac{1}{2}\rho U_{3}^{2}$$

One-dimensional models are commonly used to simulate blood flow changes, averaged blood pressure and velocity. The arterial network is segmented and connected by nodes, with each segment modeled as a compliant tube described by the axial coordinate [143]. These models are valuable for analyzing wave propagation in the vascular system and understanding pressure dynamics in a large portion of the arterial network with reasonable computational effort [23,54,58,129,145,147]. Depending on the purpose, 1D models of the CoW vary in geometric and structural complexity.

The 1D models were created by different research groups over the recent years using several solvers and input data. Regarding the latter, some studies took in vivo data as an input and modeled the geometry of the intracranial arteries [29,64,125]. Further studies considered the flow from the left ventricle to the brain-supplying arteries as input [11,59,85,114,148].

**Validation**

Olufsen et al. examined the flow in large arteries with a 1D model and compared the blood flow and pressure results with MRI measurements, which showed good agreement [62]. Huang et al. developed a 1D model, which considered the heart, lungs and neurovascular arteries. Here, the results were validated with in vivo flow data taken from the literature [61]. After normalizing the captured in vivo data, due to low resolution and variation in subjects, these could be aligned with the simulated data. Park et al. as well confirmed their results from the 1D model with in vivo blood flow data from six volunteers [63]. In vivo validation was further performed by Zhang et al. using SPECT data and by Reymond et al. using Doppler measurements [65,114]. Alastruey et al. further performed validation against in vitro data through imitating the human arteries with tubes, imitating blood with a glycerol–water mixture and measuring pressure and flow parameters [23]. Ryu et al. compared their results to the literature [66], whereas Moorhead et al. compared them to higher scale models (3D) [147].

**Application**

One-dimensional models were applied to differing variations of the CoW [23,56]. Blanco et al. analyzed a broad network with a representation of 2000 vessels and compared it to a reduced 86-vessel network [149]. Within their study, the variation in complexity showed an impact on the hemodynamics when pathologies are present. One-dimensional models were applied to pathologies like stenosis [61,150] and were widely used to examine the systemic behavior of the cerebral hemodynamics search, thus assessing the collateral capacity of the CoW. Regarding changes in the pressure and flow waveforms, Yu et al. investigated the caused changes due to aging and smoking. Alastruey et al. focused on the changes due to different pulse waves. The evaluation of pressure dynamics can be performed following these aspects: [23,54,82,119,129,147].

Moreover, a part of the application is the post-surgery analysis of the hemodynamics. One-dimensional modeling can be applied to analyze the impact of implanting a prothesis or after carotid intestinectomy [23,37]. In recent studies, also the wall modeling was considered in 1D models. Park et al. performed a precise vascular wall modeling for quantifying the interaction between the blood flow and the wall biomechanics. They compared the linear elastic model with the viscoelastic Kelvin–Voigt model and verified the data with in vivo measurements. Here, the comparison showed that when using the linear elastic model only a 4% difference was found, being even lower when the viscoelastic model was used. Regarding autoregulation, Moorhead et al. applied transient resistance at the outlets to take vasodilation and vasoconstriction into account [147]. This was also approached by Ryu et al. and Alastruey et al., who applied a resistance at the outlets, which is independent from the flow conditions in their 1D model of the aorta to the cerebrovascular vessels [30,66].

Most 1D models include simplifications and neglect wall shear stress occurrence after Womersley and use only a reduced artery system [13,35,53,151]. Herein, Formaggia et al. implemented a full body circulation without the cerebral vessels and neglected the elasticity [85]. In contrast, Reymond et al. created a full-body circuit model using a viscoelastic wall modeling of the cerebral vessels and the left ventricle [65]. Within this model, formulas for resistance and convective acceleration were included and the results show an overestimation of the pressure when compared to in vivo data [1].

Despite these wall modeling approaches, 1D models carry a disadvantage regarding the analysis of pathologies. Comprising a vascular network with reduced information, the impact of morphology and cross-sections of specific pathological vessels are not taken into account [70,119,152]. To address this concern, 3D models are proposed, especially in terms of high-resolution analysis.

### 3.3. Three-Dimensional Models

Apart from reducing the vessel geometries to a 0D or 1D structure, the inclusion of the full vessel structure is implemented within the use of 3D models. These 3D models are firstly based on the Navier–Stokes equation comprising the mass and momentum conservation equations. Secondly, they are based on on a volumetric mesh, created within on a vessel geometry and the incompressible Navier–Stokes-Equations solved on each mesh point [153].

The mass conservation is a general continuity equation as shown in Equation (12).

(12) $$\frac{\partial \rho }{\partial t}+\nabla \left(\rho u\right)=0$$

Due to incompressible flow conditions, the following formula is applied.

(13) −∇u=0

The momentum equation is a continuum equation as the mass conservation and thus described as follows:

(14) $$\rho \left(\frac{du}{dt}+u\cdot \nabla u\right)=-\nabla p+\eta \nabla^{2}u+f$$

with ρ = blood density, *u* = velocity, *p* = pressure, η = blood viscosity, *f* = body force [1].

Several assumptions have to be implemented in the modeling of computational fluid dynamic (CFD), whereas the Navier–Stokes equations are solved in a 3D space over time [82]. CFD, applied to real 3D arterial structures from clinical imaging, enables precise assessment of flow patterns and shear stresses in complex geometries. This involves modeling continuous blood flows using the mentioned continuum equations, which are then discretized into matrix equations, solved and used to obtain hemodynamic solutions.

Together with these equations, the further assumptions to compute the local flow field and depending hemodynamic parameters are described in the following.

First, blood as a non-Newtonian fluid can be modeled with the Carreau model [80,119,154,155] or the Carreau–Yasuda model to mimic the non-Newtonian behavior [13]. Nevertheless, blood is mostly handled as Newtonian fluid with constant viscosity [15,16,156]. Berg et al., Fan et al. and Razavi et al. compared the impact of the choice of fluid modeling and found no significant difference between non-Newtonian and Newtonian blood composition in laminar flows [68,73,80].

Second, the vessel walls are elastic and in a few studies modeled as such [72,76]. Fluid structure interaction (FSI) models are used to represent blood flow interactions with vessel walls in three dimensions, considering the dynamic interaction between blood as a fluid and the vessel walls [82]. The simplification has led to overestimation of wall shear stress in some local regions [82]. Several research groups considered wall thickness and found out that WSS depends on the IA’s geometry [63,157]. Moreover, FSI was applied to pathologies like wall-near thrombus formation [18] or analysis of vessel flow after brain trauma [158]. Nevertheless, in most studies focusing on cerebral vessel flow, walls are assumed to be rigid [68,159].

Third, the complex choice of the boundary condition (BC) plays an important role in 3D hemodynamic modeling [160,161]. The most realistic inlet boundary condition will for sure be the in vivo curve measured with medical imaging, as applied in recent studies [68,75,76,83]. Nevertheless, due to the usual absence of in vivo data and restrictions in data capture regarding the spatial and temporal resolution, most studies take inlet flow from sample flow measurements in human subjects or assumptions based on the literature [72,162,163]. Regarding the outlet BC, the choice is as complex as for the inlet BC, since different approaches exist and the verification is rare due to medical image restrictions. In older studies, specific blood pressure or a zero pressure condition is used [80,81,164]. In recent research, novel splitting approaches are applied to better mimic the outflow conditions [16,27,162]. Moreover, 0D resistance models are often used at the vessel outlets [1,68,117,165], which will be further examined in Section 4.2. Urquiza et al. stated that the pressure BC has an impact on the overall pulse through the vessel [132]. Therefore, a precise pressure BC is crucial to avoid velocity discrepancies due to changes in pulse.

Three-dimensional models are therefore used to compute the highly resolved neurovascular hemodynamics in complex vessel structures [1]. In particular, the impact of the morphology and prescribed boundary conditions on these hemodynamics can be analyzed using 3D models [16,166]. The geometries can be allocated into two categories [1]: idealized models, which are created artificially based on the literature [75,80,81,117,165,167], and patient-specific models, which are based on in vivo data captured with medical imaging techniques [1,13,68,72,80,83,123,125,135,168].

**Validation**

Three-dimensional models were verified using the comparison to in vivo data or to in vitro data. The latter comprise phase-contrast magnetic resonance imaging (PC-MRI) or particle-image velocimetry (PIV). Roloff et al. performed the comparison of numerically assessed IA hemodynamics to PC-MRI and PIV measurements [169] and found good agreement between the modalities. Nevertheless, they found out that within MRI the velocity can be underestimated. Brindise et al. performed a multi-modality study as well, comparing the effect of the flow field on wall shear parameters [170]. Here, non-dimensional parameters seem to provide the most stabile results. Liang et al. compared their CFD results solely to PIV [123] and received good agreement. Korte and Gaidzik et al. performed a comparison of CFD to 4D flow MRI in phantom models based on mimicking the experiments with the simulations and received comparable flow fields as well [171].

**Application**

Three-dimensional simulations were applied to physiological and pathological phenomena of particular parts of the cerebral arteries. In particular, intracranial aneurysms and vessel stenoses were taken into account in several studies [68,124,167,172]. Also, the analysis of carotid bifurcation and carotid and other variations in stenosis and IA surgery is driven from 3D CFD data [77,117,173]. Other research groups have focused on the reversed Robin Hood syndrome [165], embolic particle pathlines [72] and the simulation of contrast agent and analysis of vessel wall parameters [69]. Despite the analysis of IA hemodynamics, also the treatment effect (TE) of IAs is analysed using 3D models, which provides the opportunity for TE prediction. This virtual analysis can especially be helpful for physicians. Namely, the virtual analysis of a bypass surgery [83] or cardiopulmonary bypass procedures [78] can be analyzed. Moreover, endovascular mechanical recanalization [125] and the application of flow diverter stents were studied in the past [15,174]. The effect of devices used for IA treatment, like coil and WEB, can be studied virtually and with pre- and post-treatment analysis. Furthermore, novel devices can be tested and the efficacy shown virtually [171,175].

## 4. Multi-Scale Models

Various modeling scales have different advantages to mathematically describe the hemodynamics in the body circulation. Modeling hemodynamic interactions with vessel walls is complex, especially when analyzing pathologies like IAs and plaques, which require detailed local flow data. Three-dimensional models are ideal for these cases but depend on accurate boundary conditions. While simulating the entire arterial system in 3D would be most accurate, it is computationally infeasible. Hence, an efficient approach is to use 3D models for specific regions of interest and lower-dimensional models for the rest of the network [117]. These multi-scale models combine different spatial scales and couple 3D simulations with 0D and 1D models to introduce more realistic constraints [135].

### 4.1. Coupling 0D–1D Models

Formaggia et al. introduced a 0D–1D coupling of the descending aorta (1D) and the cardiovascular system (0D) with 30 compartments [176]. The coupling was achieved both proximally and distally at specific interfaces, ensuring a smooth transition from 0D to 1D using an iterative transitioning between time steps.

A similar algorithm was used by Pontrelli et al. to investigate the pressure distribution on the arterial wall model [177]. Liang et al. developed a closed-loop model to investigate the influence of aortic and arterial stenosis in different locations [37,86]. The utilized 1D model was built from 55 arterial parameters taken from [35,87] and incorporated elastic properties. The interface conditions allowed them to derive unknown flow and pressure variables through extrapolated Riemannian invariants, approximated via numerical iteration due to the lack of analytical solutions at interfaces. They also applied this method to study left ventricular arterial coupling in the context of age-related hemodynamic changes. The model was further extended to include the cerebral circulation and investigate the influence of the CoW anatomy on hyperfusion after carotid artery surgery.

Zhang et al. adopted a more patient-specific approach, combining 0D modeling from single-photon emission computed tomography (SPECT) data and 1D modeling from MRI data to obtain individualized hemodynamic information in the CoW [114]. The peripheral resistance of each efferent CoW artery was adjusted to match the average flow over a cardiac cycle with the reference data, minimizing peripheral resistance errors. The coupling algorithm closely resembled that of Liang et al. [37,86,90]. Validation using PC-MRI data from efferent arteries confirmed the model’s ability to capture individual variations in CoW blood flow.

Devault et al. presented a 0D–1D coupled model using Doppler ultrasound data for validation of the intracranial velocity [84]. Notably, they introduced a Kalman filter to enhance the determination of the outflow boundary condition, excluding a subset of data used for validation. Alastruey et al. also solved the 0D–1D coupled model through the Riemann problem at the bifurcations [30]. The model demonstrated that introducing a resistance equivalent to the blood vessel’s characteristic impedance at the inlet of a terminal Windkessel model is crucial for preventing the creation of unrealistic wave reflections.

Mueller and Toro et al. focused on the representation of the cardiovascular system including the venous system in the head and neck area [178]. Large cerebral arteries and veins were modeled with a 1D model and the cardiovascular and pulmonary system were modeled with 0D models. The 1D model provides the pressure terms to the 0D model so that the latter can reproduce the flow. Arterial results were validated with data from the literature and venous results were validated with PC-MRI data.

Weber et al. implemented an open-source 1D–0D solver with a 0D modeling of the cardiovascular hemodynamics and a 1D modeling of the cerebrovascular system [110]. A viscoelastic modeling of the walls was applied and parameters were based on the literature.

### 4.2. Coupling 0D–3D Models

Sun et al. coupled a closed-loop 0D model (LPM) with a 3D CoW model extracted from CT angriographic data. They focused on the analysis of stenosed arteries in the CoW and the resulting effect of stenosis formation onto CoW hemodynamics.

Yu et al. presented 3D models of the vertebral arteries based on MRI with a 0D model of the CoW. The study showed the importance of unequally distributed vertebral arteries on the magnitude of the blood flow [148].

For Lee et al., the model coupling of 0D–3D was crucial to estimate an overview of the most important aspects of the coronary and neurovascular flow reserve [179]. In the study, the limitations of simulation approaches were discussed and suggestions presented to overcome these.

Morbiducci et al. conducted a study using a CFD model to analyze the carotid bifurcation and coupled it with a vascular bed modeled as a 0D model [120]. A comparison of fixed outflow rates to a 0D model was carried out. Their findings highlighted that employing more realistic boundary conditions leads to greater accuracy in understanding the role of local hemodynamics in vascular diseases. Regarding the BCs, a fixed velocity profile is set at the inlet and a RCR network at the outlets. The coupling between the two submodels required the flow and pressures to be transferred at the interface. To overcome the complexity of the transfer between the models, in recent studies, commercial solvers and implemented application programming interfaces (API) were employed. To be mentioned here is the ANSYS Fluent Code, which was used by several researchers [32,68,120,176]. Nevertheless, due to an increased likelihood of errors in the computation when using default codes with high complexity, most researchers have preferred to use a non-extensive LP model under simplified assumptions. Moreover, the previous method limits the multi-scale model of the cardiovascular system to a single 3D model, whereas coupling two or three detailed 3D simulations with extended LP modeling of the rest of the cardiovascular system offers a greater opportunity for broader applications [117]. Kashefi et al. introduced a CAD algorithm for efficiently coupling multiple 3D models with a 0D model, substantially reducing processing time. The model was applied to investigate the carotid bifurcation, focusing on identifying regions at risk for atherosclerotic lesions. In contrast to earlier studies, within this study the Matlab toolbox Simulink was used for analyzing the 0D model, while the Fluent software was employed solely for parallel 3D computations [117].

### 4.3. Coupling 1D–3D Models

Urquiza et al. presented a 1D–3D model of the entire arterial tree, including a 3D finite element model of the carotid bifurcation coupled with a 1D model for the remaining part of the arterial tree [132]. The results were consistent with reported flow patterns in the literature and the solely executed 1D model, indicating the model’s utility in understanding the formation and development of arterial diseases.

Ho et al. used 1D and 3D models constructed based on CT images to investigate hemodynamics in IAs [122]. The resulting wall shear stress stands in agreement with the previous literature data and was validated with ultrasound measurements. Although CFD results were within an acceptable range compared to the literature, diastolic velocity was overestimated. This was traced back to the rigid wall model and therefore assumed that the use of an elastic wall model is crucial for realistic investigation of carotid artery or aortic hemodynamics [122]. However, adding elastic wall properties requires high computational power and will not affect regional parameters crucially. Thus, this is neglected in many models, especially multi-scale models, due to already high computational costs [68,80,81,164,180]. Still, the arterial wall has a high effect on pressure wave propagation throughout the vascular network. This is why Passerini et al. set the focus of their study on the effect of the wall compliance at the transfer region from the 3D to 1D model [129] using a resistor–inductor–capacitor network at the inlet and outlet.

Liang et al. modeled an anterior communicating artery aneurysm with the 1D–3D approach [123]. Based on MRI, a patient-specific 1D model with elastic walls and a 3D model of the aneurysm area with rigid walls were created. Additionally, a 1D model based on population-wide data was developed and integrated with the aneurysm model. The comparison revealed differences in the flow field and patient-specific sensitivities of hemodynamic parameters. Especially in vortex structure, in wall shear stress magnitude and distribution, and in oscillatory shear index, variations were observed. In vitro data were captured for validating the underlying results.

Sutalo et al. coupled a 3D CoW model with a 1D model, representing the peripheral cerebral vasculature [131]. They found out that stenosed peripheral vessels of the posterior cerebral artery influence the flow in vertebral arteries and of the anterior and middle cerebral arteries influence the internal carotid arteries.

### 4.4. Coupling 0D–1D–3D Models

Formaggia et al. presented an early full 0D–1D–3D coupling [176] using a 3D approach for computing the high-resolved flow inside local vessels based on medical imaging and a 1D approach to calculate the peripheral vessels based on the literature. The flow in the capillaries was numerically assessed with a 0D model, with parameters based on the literature as well and the three scale models coupled to build one system. Nevertheless, in real-world investigations, this approach was applied only in a few studies [140].

Oshima et al. applied a full-scale coupled model to different CoW anatomies to investigate the hemodynamic changes due to a missing posterior communicating artery and the free-stream outflow conditions [135]. The latter was achieved by comparing the free outflow with a multi-scale 0D/1D model for peripheral vessels. Results were compared to previous work by Tanaka et al. and Gobin et al. [181,182]. The findings showed that applying multi-scale boundary condition brought the flow closer to those calculated by the literature.

Regarding the 0D–1D–3D modeling of cerebrovascular flow, Blanco et al. investigated the flow in patient-specific intracranial aneurysms [183] and the influence of the heart rate on the flow in the carotid arteries [17]. In each case, the 3D model described the local area (blood flow), the 1D model described the systemic and peripheral arteries (wave propagation) and the 0D model described the heart, venous and pulmonary circulation (microvascular system). They achieved creating a robust model with high scalability in components and a multi-time-stepping technique to reduce the computing time.

Liang et al. investigated the pre- and postoperative patient-specific hemodynamics using a 3D model coupled with a 0D–1D cardiovascular system model [134]. The 1D submodel contains the 87 largest arteries, including in particular the 40 largest cerebral arteries, to account for hemodynamic regulation within the CoW, and the 0D submodel contains the remaining cardiovascular part, while the 3D model locally represents the middle cerebral artery bifurcation. Here as well, walls were assumed to be rigid in terms of lower computational costs. Liang et al. highlighted the importance of multi-scale models in their work by comparing them with a simple three-dimensional model. Regarding the solving of the equations, direct coupling of a rigid 3D model with an elastic 1D model leads to discontinuities of the impedance at the connection interfaces between the models. This can lead to improper wave reflections and potential numerical instability. To address these issues, a 0D interface model was set between the 1D and 3D models, based on Passerini et al. [129].

## 5. Summary and Conclusions

In this systematic review, the recent research in multi-scale modeling of the neurovascular system to describe detailed and realistic hemodynamics was presented and discussed.

**Multi-scale models as a realistic description of hemodynamic parameters within the circle of Willis**

Coupling of differently scaled models could bring the advantage of taking the full body circulation into account when analyzing neurovascular hemodynamics. Since the performance of full 3D simulation of the entire vasculature carries a high computational effort, the coupled process shows the advantage of reducing this, especially if only small regions of interest need to be investigated in detail. Up to now, detailed neurovascular hemodynamics were mostly performed using 3D simulations with assumed boundary conditions. Performing 3D simulations with patient-specific boundary conditions measured with medical imaging like PC-MRI could give the most realistic outcome. However, drawbacks also exist within these measurements due to low resolution and noise as well as non-availability.

**Advantages derived from multi-scale modeling**

If medical image data are not available or not suitable to serve as boundary conditions for 3D modeling, the use of 1D modeling as boundary condition might be beneficial. With numerical 1D modeling in contrast to medical imaging, no harm of the patient and no use of hardware resources is necessary. Moreover, using patient-specific input data for these 1D models might make them similarly realistic to the measured data [123]. In the following, if the model can be adjusted specifically to each patient, a coupled 1D–3D simulation could give a proficient representation of detailed hemodynamics in specific vessel structures. Additionally, different flow variations, pathologies and vessel formations can be analyzed comparatively using the numerical analysis without interacting with the patient [160]. This also provides the advantage of testing treatment options with regard to prediction of treatment outcomes.

**Limitations of the current status regarding multi-scale modeling**

In order to advance the progress of applying the numerical methods to the clinic, currently existing limitations in multi-scale numerical modeling of neurovascular hemodynamics should be stressed out. First to mention are the high computational time and costs, which aggravate the clinical applicability crucially due to a fast-paced clinical routine. Although the flow results are accurate and can give information about hemodynamic behavior, diagnosis prediction and treatment outcome, the processing is still time-consuming and profound statistical data is missing. Second, modeling with a high degree of detail can result in discontinuities of the coupling system which can result in non-fitting flow and pressure curves [134]. Third, a lack of validation still exists regarding multi-scale modeling. This is due to a difficulty in validation itself, which is restricted by low-resolution medical images in in vivo data and by a distance from reality in in vitro data.

**Remaining investigation needed for future improvement**

The presented mathematical and multi-scale models feature a high numerical standard to generate realistic and patient-specific hemodynamic analysis. Nevertheless, some improvements are necessary to implement these models in the clinical routine. Herein, in vivo validation is still needed to proove the applicability of the derived results, and statistical analysis should be performed, to receive significant results. Moreover, a reduction in modeling effort should be addressed to be more clinically applicable. Especially in terms of fully coupled codes, this effort could already be reduced through standardizing the coupling process. Prescribed modeling within coupling libraries can offer useful possibilities regarding a broader application [184]. These could be further addressed and implemented in future studies.

## Figures and Tables

**Table 1:** Summary of articles reviewed in this study dealing with in silico approaches to describe hemodynamics in the neurovascular and cardiovascular system in low-, high-, and multi-scales. PS: Pulmonary System; ICA: Internal Carotid Artery; MCA: Middle Cerebral Artery; ACA: Anterior Communicating Artery; TOF: Time of Flight; MRA: Magnetic Resonance Angiography; MRI: Magnetic Resonance Imaging; CT: Computed Tomography, D: dimension.

Article	D	Anatomy	Loop	Wall Model	Source of Inputdata	Software/Solver	Derived Results	Comparison
ABDI et al. (2013) [22]	0D	CoW, Heart,	closed	neglected	Literature [23]	MATLAB	Pressure–time curves	Experiments [24,25]
		PS						
BURATTINI et al. (1998) [26]	0D	Heart	open	viscoelastic	Animal study	Computer simulation	Compliance	WK2, WK3, VW, FPM models
BURKHOFF et al. (1988) [10]	0D	Aorta	open	neglected	Animal study	Computer simulation	Pressure, Impedance	Animal study
CHNAFA et al. (2017) [27]	0D	ICA, MCA,	open	neglected	3D angiograms	pyNS [28]	Flow	3D CFD results
		ACA						
CONNOLLY et al. (2014) [29]	0D	CoW	open	elastic	Literature [23,30,31]	MATLAB CVX	Flow, Pulse waveforms	Vasoconstriction to vasodilation
LAL et al. (2017) [32]	0D	CoW, Aorta	open	elastic	TOF MRA, Kalman filter	In-house	Resisistance, Compliance	Data assimilation
LI et al. (2002) [33]	0D	CoW, Heart	closed	neglected	Doppler measurements	Delphi 5.0	Pressure	Doppler measurements
MCCONNELL et al. (2017) [34]	0D	CoW	open	rigid	Literature [23,35,36]	In-house	Flow	Literature [37]
OLUFSEN et al. (2004) [38]	0D	CoW, Heart,	closed	neglected	Doppler measurements	MATLAB	Pressure, Velocity	Doppler measurements
		Full body						
POPE et al. (2009) [39]	0D	CoW, Heart	closed	neglected	Doppler measurements,	MATLAB	Pressure, Velocity	Doppler measurements
					Literature [40,41]			
SADRAIE et al. (2014) [42]	0D	CoW, Heart	open	neglected	Literature [23,43,44]	MATLAB Simulink	Pressure wave propagation	Literature [45]
WESTERHOF et al. (1969) [46]	0D	Full body	open	neglected	Literature [47,48,49]	n/a	Impedance, Pressure	Doppler measurements
ZHANG et al. (2014) [50]	0D	CoW	open	rigid	Literature [23]	n/a	Flow	Doppler measurements
ZHANG et al. (2014) [51]	0D	CoW	open	rigid	Literature [36]	Fourth-order Runge–Kutta algorithm	Flow	Doppler measurements
ALASTRUEY et al. (2007) [23]	1D	CoW, Aorta	open	elastic	Literature [35,36]	discontinuous Galerkin scheme	Flow/pressure wave propagation	Literature [52]
BESSEMS et al. (2007) [53]	1D	None	open	elastic	Galerkin	Galerkin weighted-residual method	Flow/pressure wave propagation	Womersley profiles
					weighted-residuals method			
CASSOT et al. (2000) [54]	1D	CoW	open	viscoelastic	Literature [55]	In-house	Resistance, Pressure	Non-linear numerical model
FERRANDEZ et al. (2002) [56]	1D	CoW	open	rigid	Simulated pressure	Fluent, ANSIC	Flow	Literature [57]
FITCHETT et al. (1991) [11]	1D	CoW, Heart,	closed	viscoelastic	Numerically assessed	n/a	Pressure, Stroke work,	n/a
		Full body					Impulse reponse	
FORMAGGIA et al. (2003) [58]	1D	Endograft	open	elastic	Pressure waveforms	Finite element Taylor–Galerkin scheme	Flow, Pulse waveforms	2D fluid–structure interaction
HUANG et al. (2015) [59]	1D	CoW, Full body	closed	elastic	n/a	TVD scheme	Velocity, Pressure, Flow	Literature [60]
HUANG et al. (2018) [61]	1D	CoW	open	elastic	MRI/CT measurements	THINkS	Flow, Velocity	Literature [60]
OLUFSEN et al. (2000) [62]	1D	Full body	open	elastic	MRI measurements	Two-step Lax–Wendroff method	Algorithms, Pressure	MRI measurements
PARK et al. (2019) [63]	1D	CoW	open	elastic	MRI measurements	Method of weighted residuals	Pressure, Flow, Area	MRI measurements
						Fourier series		
PERDIKARIS et al. (2015) [64]	1D	CoW, Arm	closed	viscoelastic	MRI/PCMRI measurements	Discontinuous Galerkin solver	Pressure, Flow	n/a
REYMOND et al. (2009) [65]	1D	CoW, Heart,	open	viscoelastic	MRI/Doppler measurements,	Witzig–Womersley Theory	Pressure, Flow, Area	MRI/Doppler measurements,
		Full body			Tonometry			Tonometry
RYU et al. (2015) [66]	1D	CoW, Heart	open	elastic	Literature [23,35,36,67]	Second-order spatial scheme	Flow velocity, Activation factor,	Simulations
						Two-step Adams–Bashforth temporal scheme	Active tension, Flow	
BERG et al. (2014) [68]	3D	CoW	open	rigid	MRI measurements	ANSYS Fluent	Velocity fields, Angular similarity index,	n/a
							Magnitude similarity index	
CEBRAL et al. (2003) [69]	3D	CoW	open	rigid	MRI/PCMRI measurements	In-house simulation	Flow, Resistances,	n/a
							Shear parameters (OSI, WSS)	
CHAICHANA et al. (2011) [70]	3D	Coronary arteries	open	rigid	Literature [71]	ANSYS CFX, MATLAB	WSS, WSS gradient, Velocity	n/a
FABBRI et al. (2014) [72]	3D	CoW	open	rigid	MRI measurements	ANSYS CFX, Particle–fluid interaction	Particle tracking	n/a
FAN et al. (2009) [73]	3D	Carotid	open	rigid	Literature [74]	FLUENT, GAMBIT	Velocity, WSS	n/a
GHAFFARI et al. (2017) [75]	3D	CoW	open	rigid	MRI measurements,	ANSY Fluent	Morphology, Wall shear,	Statistical analysis
					NOVA scans		Vorticity, Pressure, Flow	
GRINBERG et al. (2011) [76]	3D	CoW	open	rigid	PC-MRI measurements	High-order spectral/hp element solver	Pressure, Flow distribution	n/a
MAMATYUKOV et al. (2018) [77]	3D	CoW	open	elastic	Doppler measurements	ANSYS CFX	Velocity	n/a
PISKIN et al. (2015) [78]	3D	CoW	open	rigid	Literature [79]	ANSYS Fluent	Velocity, WSS, Pressure	Literature [79]
RAZAVI et al. (2014) [80]	3D	CoW	open	rigid	MRI measurements	COMSOL	Velocity, Flow, Shear rate	n/a
REN et al. (2015) [81]	3D	CoW	open	rigid	MRI/PCMRI measurements	ANSYS Fluent	Velocity, Flow	n/a
ZHANG et al. (2020) [82]	3D	CoW	open	rigid	MRI measurements	ANSYS CFX	Velocity, Shear parameters	n/a
ZHU et al. (2018) [83]	3D	CoW	open	rigid	MRI/PCMRI measurements,	ANSYS CFX	Pressure drop, WSS, Flow	NOVA data
DEVAULT et al. (2008) [84]	0D-1D	CoW	open	viscoelastic	MRI measurements	Chebyshev collocation methods	Velocity, Pressure, Flow	Transcranial Doppler
FORMAGGIA et al. (2006) [85]	0D-1D	Heart and arteries	open	viscoelastic	Blood pressure measurements	Second-order Taylor–Galerkin FE scheme	Flow, Pressure	n/a
LIANG et al. (2009) [86]	0D-1D	CoW, Full body	closed	elastic	Literature [35,87,88]	Two-step Lax–Wendroff method	Blood flow, Pressure	Literature [89]
						Fourth-order Runge–Kutta method	Stroke volume	
LIANG et al. (2009) [90]	0D-1D	CoW, Full body	closed	elastic	Literature [35,87,88]	Two-step Lax–Wendroff method	Blood flow, Pressure	n/a
						‘Ghost- point’ method	Stroke volume	
LIANG et al. (2011) [37]	0D-1D	CoW, Heart	closed	elastic	In vivo data, Literature [23,65,84]	Two-step Lax–Wendroff method	Flow, Hyperfusion	In vivo data,
						Fourth-order Runge–Kutta method		Literature [23,65,84]
MÜLLER et al. (2014) [60]	0D-1D	Total	closed	elastic	Literature [37]	Dumbser–Enaux–Toro (DET) solver	Pressure, Flow	MRI measurements,
								Literature [91,92,93,94,95,96,97,98,99,100,101,102,103,104,105,106,107,108,109]
WÉBER et al. (2023) [110]	0D-1D	CoW, Heart,	open	Poynting–	Literature [65,111,112]	C++	Pressure, Flow	Literature [113]
		Full body		Thomson				
ZHANG et al. (2016) [114]	0D-1D	CoW, Heart,	closed	elastic	In-house software „V-modeler“ [115],	In-house simulation	Flow	Literature [23,35,65,116]
		Full body			MRI/PC-MRI measurements,			
					SPECT data			
KASHEFI et al. (2014) [117]	0D-3D	Carotid, Heart,	open	elastic	Literature [74]	Matlab Simulink, ANSYS Fluent	Velocity distribution,	Steady simulation [118]
		Full body					Pressure, Flow	
LIU et al. (2016) [119]	0D-3D	CoW, Aorta	open	rigid	MRI/Doppler measurements	Comsol Multiphysics	Velocity, Flow	Doppler measurements
MORBIDUCCI et al. (2010) [120]	0D-3D	Carotid	open	rigid	CTA (angiographic images)	ANSYS Fluent	Wall shear parameters	n/a
SUN et al. (2023) [121]	0D-3D	CoW	closed	rigid	CTA	ANSYS CFX		
HO et al. (2009) [122]	1D-3D	CoW	open	rigid	CTA	ANSYS CFX	Velocity	Doppler measurements
LIANG et al. (2016) [123]	1D-3D	CoW, IA	open	rigid	MRI measurements	In-house code, Two-Step Lax-Wendroff method	Velocity, Flow, OSI, WSS	PIV measurements
MARZO et al. (2011) [124]	1D-3D	CoW	open	elastic	3DRA/PC-MRI measurements	ANSYS CFX	Flow, Shear Parameters	n/a
NEIDLIN et al. (2016) [125]	1D-3D	CoW	open	rigid	CTA	ANSYS CFX	Flow, Velocity	Literature [126,127,128]
PASSERINI et al. (2009) [129]	1D-3D	CoW, Carotid	open	rigid	Literature [23,30,130]	LifeV	Flow, Velocity	n/a
ŠUTALO et al. (2014) [131]	1D-3D	CoW	open	elastic	CT	ANSYS CFX	Flow, Pressure, Velocity	n/a
URQUIZA et al. (2006) [132]	1D-3D	Full body	open	elastic	Literature [74]	SUPG [133]	Flow, Velocity, Shear stress	Literature
BLANCO et al. (2010) [17]	0D-1D-3D	Full body, Aorta	closed	viscoelastic	MRI measurements	Gauss–Seidel method	Flow, OSI, Particle tracking	n/a
LIANG et al. (2015) [134]	0D-1D-3D	CoW, Full body	open	rigid	Literature [86,90]	Two-step Lax–Wendroff method	WSS, Flow, Velocity	3D simulation
						Fourth-order Runge–Kutta		
OSHIMA et al. (2012) [135]	0D-1D-3D	CoW	open	rigid	MRI measurements	In-house	WSS, Pressure, Flow	n/a

## Data Availability

Not applicable.

## Abbreviations

The following abbreviations are used in this manuscript:

A	Cross-Sectional Area
C	Capacitance
CFD	Computational Fluid Dynamics
CoW	Circle of Willis
CT	Computed Tomography
D	Dimensional
FSI	Fluid Structure Interaction
I	Current
IA	Intracranial Aneurysm
$K_{R}$	Blood Resistance
L	Inductance
LPM	Lumped Parameter Model
MRI	Magnetic Resonance Imaging
ODE	Ordinary Differential Equation
P	Pressure
PC	Phase Contrast
PDE	Partial Differential Equation
PIV	Particle-Image Velocimetry
Q	Flowrate
R	Resistance
SPECT	Single-Photon Emission Computed Tomography
t	Time
U	Voltage
WK	Windkessel
z	Vessel Length
